# Knockdown of mitofilin inhibits autophagy and facilitates starvation-induced apoptosis in HeLa cells

**DOI:** 10.22038/ijbms.2019.36173.8617

**Published:** 2019-10

**Authors:** Mengli Shen, Li Wang, Lingyun Kuang, Danhui Liu

**Affiliations:** 1 Zhejiang Provincial Key Laboratory of Medical Genetics, Key Laboratory of Laboratory Medicine, Ministry of Education, School of Laboratory Medicine and Life Sciences, Wenzhou Medical University, Wenzhou, Zhejiang, China 325035

**Keywords:** Apoptosis, Autophagy, HeLa cell, Mitofilin, Starvation

## Abstract

**Objective(s):**

Mitofilin contributes to the maintenance of mitochondrial structure and functions. This study was undertaken to determine the mechanisms underlying its regulation of apoptosis.

**Materials and Methods:**

Mitofilin was knockdowned by specific short hairpin RNA (shRNA) and the stable HeLa cell clone was selected. The autophagy activity were assessed with LC3-II conversion and puncta formation by western blot and fluorescence imaging in starved and normal cultured HeLa cells. Autophagy flux was measured in the presence of NH_4_Cl. Wortmannin was used to inhibit autophagy. Cell viability and apoptosis were detected with cell counting kit-8 (CCK-8) and fluorescence-activated cell sorting (FACS) assay, respectively.

**Results:**

Mitofilin expression was down-regulated in starved HeLa cells. In established mitofilin stable knockdown cell lines, LC3-II conversion and puncta formation were detected, which are both hallmarks of autophagy, under both basal and starvation conditions. Mitofilin down-regulation decreased LC3-II conversion and puncta formation, which indicates that loss of mitofilin function inhibits both basal and starvation-induced autophagy activity. CCK-8 and FACS analysis confirmed mitofilin involvement in the regulation of cell survival since mitofilin down-regulation facilitated starvation-induced apoptosis in HeLa cells.

**Conclusion:**

Taken together, mitofilin is a potent regulator of autophagy and it may modulate cell survival through regulation of autophagy.

## Introduction

Autophagy is a catabolic process essential for important physiological processes such as development, differentiation, cell survival and cellular homeostasis (1). The most well-known variant of autophagy is macroautophagy (herein referred to as autophagy). In this process, cytoplasmic materials are sequestered by double-membraned autophagosomes (1). Autophagosomes then fuse with lysosomes to hydrolyze the cargo, which can be reused by the cells (1). Low level basal autophagy is very important in eliminating the byproducts of normal cellular processes, which is essential for preserving the physiological homeostasis (2). Autophagy can also be induced by the fluctuations of intracellular and extracellular microenvironment such as metabolic, oxidative and nutritional stresses (2). These autophagic responses are critical for the maintenance of normal cell functions. 

The role of autophagy in tumor development has been extensively explored. Autophagy emerges as a tumor suppression mechanism since essential autophagy gene ATG6/Beclin1 is often monoallelically lost in prostate, breast and ovarian cancers (3, 4). Moreover, Beclin1 deficiency promotes tumorigenesis in mice (5, 6). On the contrary, however, autophagy may also play a pro-survival role in tumor cells starved by inadequate blood supply, which suppresses tumor initiation and invasion (7). Generally, cancer cells rely more on autophagy than normal cells due to their nutrient deficient microenvironment and increased metabolic demands needed to support robust proliferation (8). In addition, there is evidence showing that autophagy may protect tumor cells during anticancer therapy (9). 

In an effort to find potential regulator of starvation-induced autophagy, we found that the expression of mitofilin is down-regulated in starved HeLa cells. Mitofilin, also designated IMMT, is an inner mitochondrial membrane protein. It is a central component of the mitochondrial inner membrane organizing system (MINOS) complex, which includes also SAM50, metaxin 1 and 2, CHCHD3, CHCHD6, DnaJC11, C1orf163, APOOL and other proteins (10). Decreased expression of mitofilin has been observed in many human diseases such as Down’s syndrome, Parkinson’s disease, epilepsy, type 1 diabetes, and neurodegeneration (11-13). These results clearly show that mitofilin has critical physiological roles.

The importance of mitofilin in the maintenance of mitochondrial structure and function has been well-defined. Knockdown of mitofilin blocked formation of mitochondrial cristae junctions, increased the production of reactive oxygen species, depolarized the membrane potential difference, and decreased ATP production (14). Transgenic expression of mitofilin preserves mitochondrial structure and restores mitochondrial function in the diabetic heart (15). Mitofilin expression is also involved in the regulation of tumor cell death. Down-regulation of mitofilin resulted in the fragmentation of the mitochondrial network, disorganization of cristae and enhanced release of cytochrome c during apoptosis in HeLa cell (16). It has been shown that knockdown of mitofilin induces apoptosis and cell cycle arrest through modulation of the apoptosis-inducing factor- poly (ADP-ribose) polymerase (AIF-PARP) signaling pathway (17). 

Mitochondria are the center of cellular energy production and play important roles in the regulation of autophagy (18). It is reasonable that mitofilin may regulate autophagy considering its important roles in mitochondrial maintenance. In fact, it has been shown that phosphorylation of mitofilin by protein kinase A (PKA) regulates PTEN-induced kinase 1 (PINK1) stability and Parkin recruitment to damaged mitochondria, which is an essential process modifying selective mitochondrial autophagy (19, 20). However, whether mitofilin regulates autophagy still remains unknown. Based on the context, we hypothesized that mitofilin regulates cell death through modulation of autophagy. 

In this report, we describe the functional contribution of mitofilin in modulating autophagy activity in HeLa cells. Such an assessment entailed evaluating the effect of loss of its function on autophagy activity under basal and starvation level. Moreover, we tried to elucidate the potential role of mitofilin-regulated autophagy in modulating cell viability and apoptosis. 

## Materials and Methods

***Cell culture and transfection***

HeLa cells were purchased from American Type Culture Collection (ATCC; Manassas, VA). Cells were cultured in Dulbecco’s modified Eagle’s medium (DMEM, Invitrogen, Carlsbad, CA) with 10% fetal bovine serum (FBS; Hyclone, Logan, UT) and 1% penicillin-streptomycin (Invitrogen) at 37°C in 5% CO_2_. Short hairpin RNA (shRNA) for mitofilin (5’-GCTAAGGTTGTATCTCAGTAT-3’) and its negative control were cloned into pGPU6/Neo (Genepharma, shanghai, China). To establish stable cell lines, HeLa cells were transfected and then selected with 1 mg/ml neomycin. 

For starvation-induced autophagy, HeLa cells were cultured in Hank’s balanced salt solution (HBSS, Invitrogen) for 2 hr. 100 nM wortmannin (Sigma, St. Louis, MO) was used to inhibit autophagy. To measure the autophagy flux, HeLa cells were pretreated with 20 mM NH_4_Cl (Sigma), and then LC3 conversion and punctuation were analyzed. 

**Figure 1:**
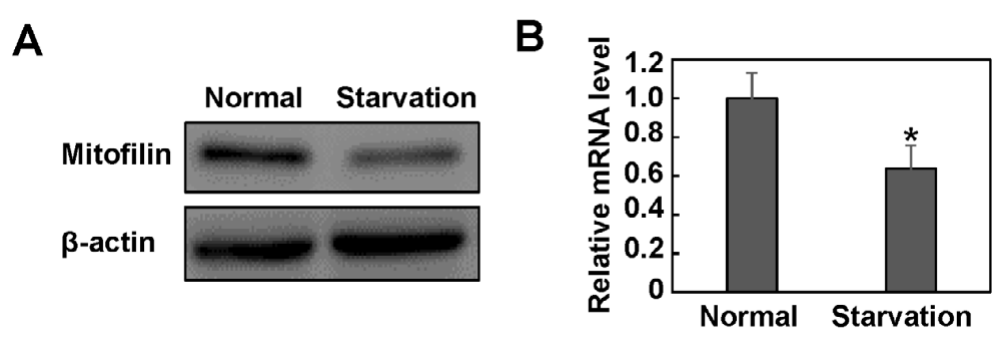
Mitofilin is down-regulated in starved HeLa cells. HeLa cells were cultured in Hank’s balanced salt solution for 2 hr. The cell lysate and total RNA were extracted and subjected to western blotting (A) and real-time PCR assay (B). β-actin was detected as a loading control in immunoblot. The mRNA level of mitofilin was normalized to the level of β-actin. Images are representative of three independent experiments. Data are expressed as mean±SD. * P<0.05

**Figure 2:**
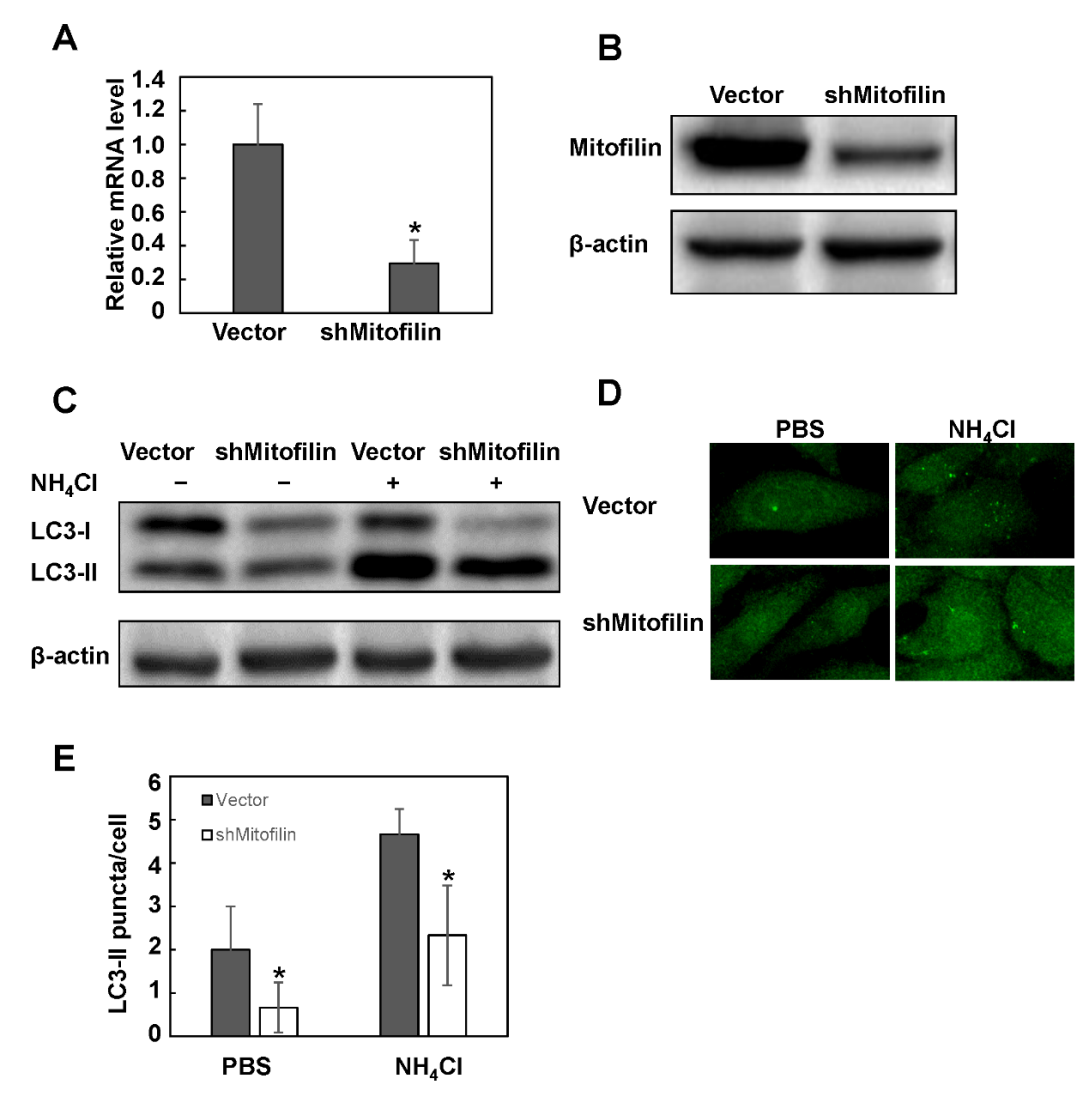
Knockdown of mitofilin inhibits basal autophagy activity. HeLa cells were transfected with mitofilin specific short hairpin RNA (shRNA) and selected with G418. Mitofilin expression level in the established stable cell line was detected at mRNA (A) and protein (B) expression levels. β-actin was used as internal control. (C) The LC3-II conversion was detected with western blotting. For the measurement of autophagy flux, cells were pretreated with 20 mM NH_4_Cl. (D) The LC3-II puncta distribution was assessed with immunofluorescence (400X), and (E) the LC3 puncta per cell were counted. Pretreatment of NH_4_Cl was used to evaluate the autophagy flux. Images are representative of three independent experiments. Data are expressed as mean±SD. * *P*<0.05

**Figure 3:**
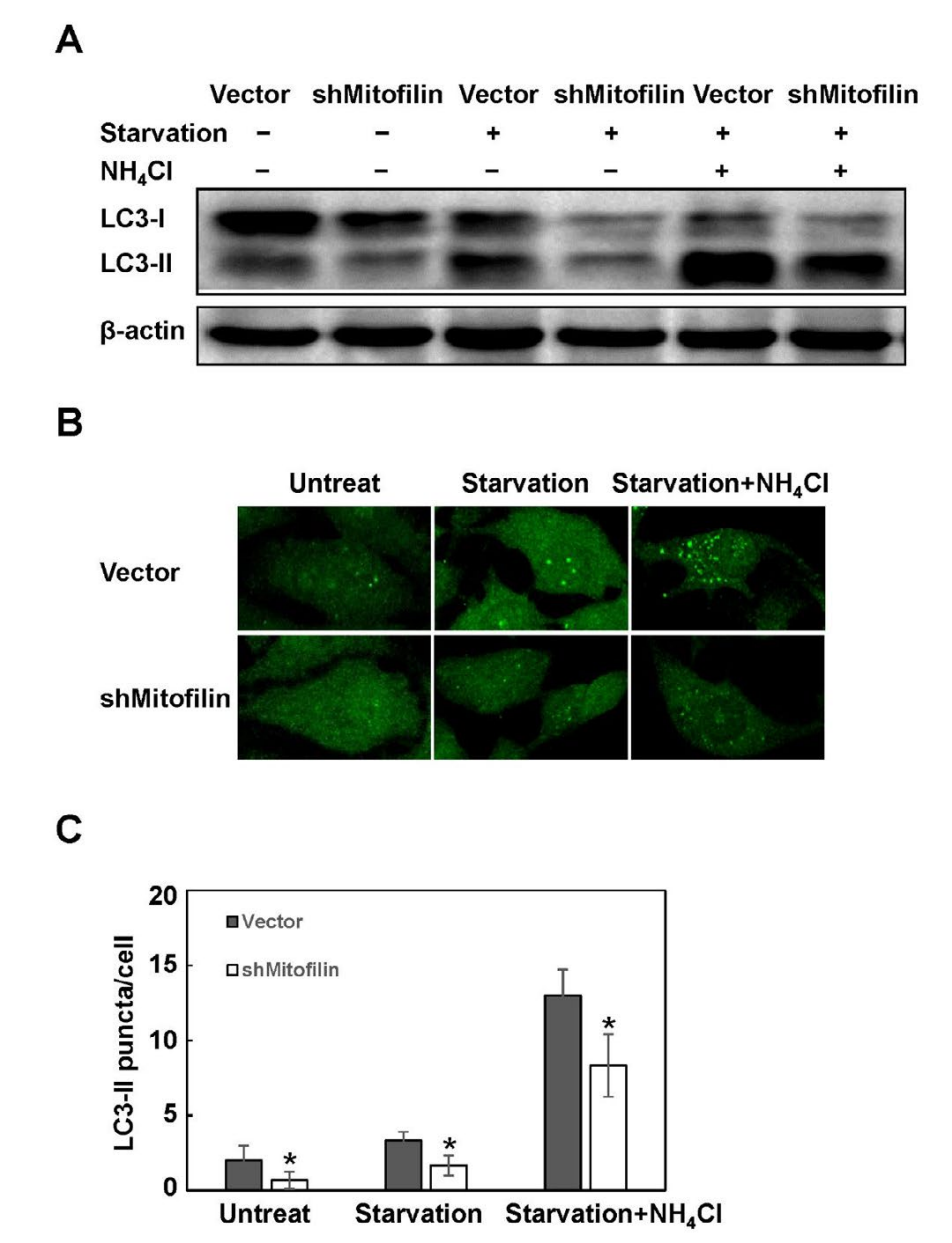
Mitofilin down-regulation inhibits starvation-induced autophagy. Mitofilin knockdown cells and vector cells were starved for 2 hr, and the LC3 conversion and puncta formation were detected with immunoblot (A) and immunofluorescence (B, 400×). NH_4_Cl was used for the assessment of autophagy flux. (C) The LC3 puncta were counted. β-actin was used as internal control. Images are representative of three independent experiments. Data are expressed as mean±SD. * *P*<0.05

**Figure:**
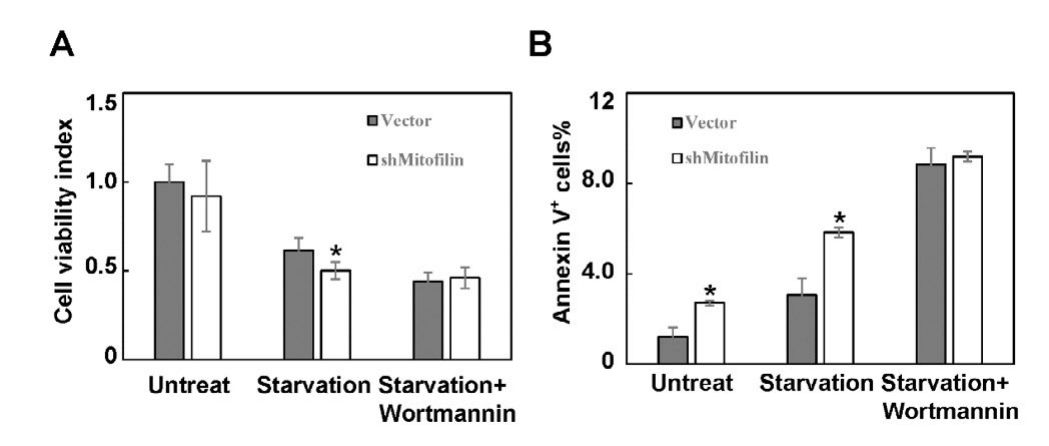


***RNA exaction and real-time PCR***

Total RNA was extracted with Trizol reagent (Invitrogen) and cDNA was prepared according to manufacturer’s instruction. Primers used for mitofilin detection were: sense: 5‘- TGAGATTGCAGGCGAGAA-3’, antisense: 5’- CTTTGAGAAGGGCATCGG-3’. For β-actin: sense: 5’- ACCCACACTGTGCCCATCTAC-3’, antisense: 5’- TCGGTGAGGATCTTCATGAGGTA-3’. The quantification of mRNA levels was performed using SYBR Green dye (Invitrogen), and the relative mRNA quantitation was performed using the comparative Ct method. The melting curve analysis confirmed that only a single amplicon was generated by qPCR (supplemented Figure 1). All the data were normalized to the level of β-actin mRNA.

***Cell lysates and western blot analysis***

HeLa cells were harvested with trypsin (Invitrogen) and lysed with lysis buffer (50 mM Tris-HCl, 1 mM EDTA, 20 g/L sodium dodecyl sulfate (SDS), 5 mM dithiothreitol, and 10 mM phenylmethylsulfonyl fluoride). 20 μg cell lysates/well along with the protein marker were separated on 12% SDS-PAGE gels and transferred onto nitrocellulose membranes. The membranes were blocked with 5% nonfat milk in phosphate-buffered saline (PBS) with 0.05% Tween-20 for 2 hr at room temperature, and then incubated with the primary antibody overnight at 4 ^°^C. After washing with PBS buffer containing 0.05% Tween-20, the membranes were incubated with HRP-conjugated secondary antibody and then detected with enhanced chemiluminescence detection kit (Pierce, Rockford, IL). β-actin was detected as loading control. Primary antibodies for mitofilin, LC3, and β-actin were purchased from Abcam. The secondary antibodies were from Santa Cruz. 

***Immunofluorescence staining***

Cells were seeded on the cover slides and treated as indicated. Cells were then fixed with 4% paraformaldehyde solution for 1 hr at room temperature and permeabilized with 0.4% Triton X-100 in PBS for 10 min. The cells were blocked using 5% BSA in PBS for 1 hr at room temperature and then incubated with LC3 primary antibody overnight at 4 ^°^C. After washing with PBS, cells were then incubated with Cy3-conjugated secondary antibody for 1 hr at room temperature. Cells were washed with PBS and the nuclei were stained with 4’,6-diamidino-2-phenylindole (DAPI). Finally, cells were mounted with anti-fade mounting medium and observed under a fluorescence microscope. 

***Cell viability and cell apoptosis analysis***

Cell counting kit-8 (CCK-8, Sigma) was used to assess the cell viability according to the manufacturer’s protocol. Briefly, HeLa cells were seeded in 96-well microplate and treated as indicated. CCK-8 working solution was added to each well and incubated for 2 hr at 37 ^°^C. The reactions were stopped by adding 1% SDS. Cell viability was evaluated by measuring the absorbance at 450 nm and subtracting the absorbance at 600 nm with a microplate reader. 

Cell apoptosis was assessed by flowcytometry. Treated HeLa cells were stained with fluorescein isothiocyanate (FITC)-conjugated Annexin V to detect the phosphatidylserine externalization, one of the hallmarks of apoptosis. And propidium iodide (PI) was used to stain the nuclei. Cells were then analyzed by flowcytometry using a FACSCalibur (BD biosciences). 

***Statistical analysis***

All experiments were carried out at least three times. All data were shown as mean ± SD. Differences between data groups were calculated using the Student’s t-test. *P *values<0.05 were considered statistically significant. 

## Results

***Mitofilin is down-regulated in starved HeLa cells***

To determine if mitofilin contributes to the regulation of autophagy, we detected the expression of mitofilin in starved HeLa cells. Starvation is a well-accepted direct method to induce autophagy. The western blotting results showed that the expression of mitofilin was decreased after 2 hr of starvation, whereas the β-actin level was not affected (Figure 1A). We further examined the mRNA level of mitofilin, and the real-time PCR results showed that the mitofilin mRNA level dropped 36.3% in starved HeLa cells compared to untreated cells (Figure 1B). This decline suggests that starvation suppresses mitofilin expression at least partly through inhibiting its transcription.

***Knockdown of mitofilin inhibits the baseline autophagy activity***

To determine if mitofilin is involved in the regulation of autophagy, we used shRNA to knockdown its expression and established stable cell line. The western blotting and real-time PCR results showed that the mitofilin level was significantly reduced at both protein and mRNA expression levels (Figure 2A, B). The microtubule-associated protein 1A/1B ligh china 3(LC3) conversion and the formation of LC3-II puncta are hallmarks of autophagy (21). The immunoblot results and band density quantification results showed that knockdown of mitofilin decreased the level of LC3-II compared to the empty vector group (Figure 2C and data not shown). We further assessed the autophagy flux in the presence of lysosome inhibitor NH_4_Cl. The results showed that the autophagy flux was much less in mitofilin knockdown cells than in the control group (Figure 2 C). These results suggest that mitofilin knockdown inhibited the formation of autophagosome. To further confirm the result, we detected the distribution of LC3-II. Knockdown of mitofilin decreased the LC3-II puncta number in HeLa cells. Treatment of NH_4_Cl increased the puncta number, and knockdown cells still showed fewer puncta than the control cells (Figure 2D, E). Taken together, these data suggest that knockdown of mitofilin inhibits the baseline autophagy activity in HeLa cells. 

***Knockdown of mitofilin inhibits starvation-induced autophagy***

Since knockdown of mitofilin inhibited the baseline autophagy, we assumed that it could also block starvation-induced autophagy. Indeed, subjecting mitofilin-knockdown cells to starvation decreased the LC3-II level in the absence or presence of NH_4_Cl (Figure 3A). In the immunofluorescence assay, starvation increased the LC3-II puncta number, but fewer puncta were observed in the mitofilin-knockdown cells. Treatment with NH_4_Cl increased the number of both vector and mitofilin-knockdown cells. Nevertheless, the number of knockdown cells was still less than that in the empty vector group (Figure 3B,C). These data indicate that mitofilin modulates starvation-induced autophagy activity.

***Down-regulation of mitofilin facilitates starvation-induced apoptosis***

Autophagy plays an important role in cell survival during tumor initiation when they are deprived of adequate nutritional support. Since mitofilin down-regulation inhibits both baseline and starvation-induced autophagy in HeLa cells, we further investigated if mitofilin regulates HeLa cell survival. CCK-8 assay was used to evaluate the cell viability. Mitofilin knockdown by itself did not cause obvious reduction in cell viability (Figure 4A). However, in starved mitofilin-knockdown cells, the cell viability was significantly decreased compared to the control group (Figure 4A). In the presence of wortmannin, an autophagy inhibitor, cell viability further decreased to similar level in both normal and starved groups (Figure 4A). This result suggests that mitofilin may play a pro-survival role in starved HeLa cells. We performed Annexin V fluorescence-activated cell sorting (FACS) analysis to further evaluate if mitofilin modulates apoptosis. The results showed that the number of Annexin V^+^ cells was a little higher in mitofilin knockdown cells than in the control group (Figure 4B). Starvation treatment increased the apoptosis rate in both vector group and mitofilin knockdown group, whereas the apoptosis rate in the knockdown cells was still much higher than that in the empty vector group. Finally, treatment with wortmannin augmented the number of apoptotic cells in both groups to a similar level (Figure 4B). Taken together, these results suggest that mitofilin knockdown enhanced starvation-induced apoptosis through inhibition of autophagy.

## Discussion

The importance of mitofilin in the maintenance of mitochondrial structure and function has been extensively explored. Mitofilin-regulated maintenance of cristae structure integrity is dose-dependent (16). A 95% loss in mitofilin expression will cause severely abnormal onion-like mitochondrial cristae structure, whereas a less mitofilin loss forms vesicle-like cristae with increased intermembrane space and thus may allow cytochrome c release, which is an important initiation step of intrinsic apoptosis pathway (16). These structural deficits caused by the loss of mitofilin indicate that it is a regulator of apoptosis. Indeed, Ngonidzashe *et al*. showed that knockdown of mitofilin induces apoptosis and cell cycle arrest through modulation of the AIF-PARP signaling pathway (17). Similarly, Van *et al.* found that shRNA-mediated knockdown of mitofilin potentiated dopamine-induced cell death in SH-SY5Y and PC12 dopaminergic cell lines (22). Conversely, mitofilin overexpression inhibited dopamine- and rotenone-mediated cell death (22). 

In an effort to determine if mitofilin is a regulator of autophagy, we first showed that mitofilin expression was down-regulated in starved HeLa cells. To further explore the physiological relevance of this reduction, we analyzed the autophagy activity in mitofilin knockdown cells. LC3-II conversion and accumulation were detected with western blotting and immunofluorescence and the autophagy flux was measured in the presence of 20 mM NH_4_Cl. All of the data showed that mitofilin contributes to the regulation of autophagy. To the best of our knowledge, this is the first report documenting that mitofilin is a regulator of autophagy.

Autophagy is a conserved metabolic process that is essential for many physiological processes, and dysregulated autophagy is known to be involved in many human diseases such as neurodegeneration, tumorigenesis, aging and Parkinson’s disease (23). Cells use autophagy to orderly degrade cellular components and reuse the products such as amino acids, lipids and carbohydrates (24). Low level basal autophagy eliminates damaged organelles and byproducts of normal cellular processes. This is essential for the maintenance of cellular homeostasis. Basal autophagy is usually higher in tumor cells due to a nutrient deficient microenvironment resulting from increased metabolic demands by more robust proliferation (24). Moreover, autophagy plays pro-survival role in starved tumor cells due to an inadequate blood supply, which is insufficient to support tumor initiation and invasion (7). Moreover, tumor cells rely on autophagy to degrade apoptosis mediators. The combined use of autophagy inhibitors and antineoplastic drugs enhances the effectiveness of anticancer therapies (25). After demonstrating that mitofilin is a regulator of autophagy, we showed that mitofilin-modulated autophagy plays a role in the regulation of cell survival. CCK-8 assay results showed that knockdown of mitofilin markedly reduced cell viability. FACS results further demonstrated that mitofilin down-regulation facilitates starvation-induced apoptosis in HeLa cells. Treatment with wortmannin, an autophagy inhibitor, further potentiated apoptosis to similar level in mitofilin knockdown cells and vector group. These results suggest that mitofilin modulates starvation-induced apoptosis through regulation of autophagy in HeLa cells. 

Mitofilin is down-regulated in many human diseases such as Down’s syndrome, Parkinson’s disease, epilepsy, type 1 diabetes, and neurodegeneration (11-13). The current results provide evidence that mitofilin-modulated autophagy may play a role in the progression of these different diseases since dysregulation of autophagy is a contributing factor in their pathophysiology. The underlying mechanisms of mitofilin-regulated autophagy remain unknown. Mitofilin contributes to preserving mitochondrial structural integrity, which is critical for preserving its function. This requirement is evident because declines in its expression accompany losses in mitochondrial structural integrity which may, in turn, induce apoptosis due to leakage of cytochrome c into the cytosol and caspase activation (17).  Such changes could alter the machinery needed for supporting autophagy because Beclin1, PI3K, and ATG4D are all substrates of caspases (26, 27). In other words, the mitofilin expression is essential for maintaining a homeostatic balance between autophagy and apoptosis. Besides, there are reports showing that mitochondrial dysfunction seemed to inhibit phagosomal initiation and lysosomal acidification in mammalian cells (28). Thus, a pathophysiological condition that disrupts mitochondrial structural integrity could disrupt this balance between autophagy and apoptosis.

## Conclusion

Taken together, mitofilin is down-regulated in starved HeLa cells. Knockdown of mitofilin inhibits both basal and starvation-induced autophagy, and thus augments starvation-induced apoptosis. The detailed mechanisms accounting for how mitofilin modulates autophagy require further study. 
